# The Transcriptional Foundations of Sp110-mediated Macrophage (RAW264.7) Resistance to *Mycobacterium tuberculosis* H37Ra

**DOI:** 10.1038/srep22041

**Published:** 2016-02-25

**Authors:** Yongyan Wu, Zekun Guo, Kezhen Yao, Yue Miao, Shuxin Liang, Fayang Liu, Yongsheng Wang, Yong Zhang

**Affiliations:** 1 College of Veterinary Medicine, Northwest A&F University, Yangling 712100, Shaanxi, China; 2 Key Laboratory of Animal Biotechnology, Ministry of Agriculture, Northwest A&F University, Yangling 712100, Shaanxi, China; 3 Innovative Experimental College, Northwest A&F University, Yangling 712100, Shaanxi, China

## Abstract

Human tuberculosis (TB), caused by *Mycobacterium tuberculosis* (*Mtb*), remains a leading global health problem, causing 1.3 million deaths each year. The nuclear body protein, Sp110, has been linked to TB resistance and previous work showed that it enhances macrophage apoptosis upon *Mtb* infection. Here, we report on the role of Sp110 in transcriptional regulation of macrophage responses to *Mtb* through integrated transcriptome and mechanistic studies. Transcriptome analysis revealed that Sp110 regulates genes involved in immune responses, apoptosis, defence responses, and inflammatory responses. Detailed investigation revealed that, in addition to apoptosis-related genes, Sp110 regulates cytokines, chemokines and genes that regulate intracellular survival of *Mtb*. Moreover, Sp110 regulates miRNA expression in macrophages, with immune and apoptosis-related miRNAs such as miR-125a, miR-146a, miR-155, miR-21a and miR-99b under Sp110 regulation. Additionally, our results showed that Sp110 upregulates BCL2 modifying factor (Bmf) by inhibiting miR-125a, and forced expression of Bmf induces macrophage apoptosis. These findings not only reveal the transcriptional basis of Sp110-mediated macrophage resistance to *Mtb*, but also suggest potential regulatory roles for Sp110 related to inflammatory responses, miRNA profiles, and the intracellular growth of *Mtb*.

Human tuberculosis (TB), caused by the intracellular pathogen *Mycobacterium tuberculosis* (*Mtb*), remains a leading global health problem^1^. Concurrently, bovine TB caused by *Mycobacterium bovis* (*M. bovis*) continues to be a major problem in cattle and poses a threat to human health^2^. Macrophages are the main host cells for mycobacteria, and infection with mycobacteria induces macrophage apoptosis or necrosis. It has been shown that virulent mycobacteria have evolved to evade host defence responses by inducing macrophage necrosis, but inhibiting macrophage apoptosis^3^. Mycobacteria also alter host gene expression to modulate host immunity. *M. bovis* bacillus Calmette–Guérin infections induce IFN-γ production by inhibiting miR-29 expression^4^. H37Rv upregulates miR-99b expression in murine dendritic cells and macrophages, thereby decreasing expression of the miR-99b targets, Tnf and Tnfrsf4^5^. Accordingly, cytokine expression in the cells is altered, and this affects activation of the host immune response and the survival of intracellular mycobacteria.

Approximately one-third of the global human population is infected with *Mtb*, making eradication of TB difficult to achieve. However, only 10% of latent infections develop into active TB, indicating that inherited factors and host immune defences can affect the outcome of *Mtb* infections^6^. The susceptibility to TB 1 (*sst1*) locus on mouse chromosome 1 controls the progression of tuberculosis^7^. Subsequent studies have identified a candidate gene within the *sst1* locus designated as intracellular pathogen resistance 1 (also known as *Sp110*), and this gene regulates innate immunity to infection with *Mtb*^8^. Overexpression of Sp110 enhances apoptosis and inhibits necrosis of mycobacterium-infected macrophages^8^,^9^. Recent studies have focused on associations between *SP110* gene polymorphisms and tuberculosis susceptibility^10^,^11^,^12^. However, the molecular mechanisms of Sp110-mediated macrophage resistance to *Mtb* remain unknown.

Recently, we generated transgenic cattle that overexpress the mouse *Sp110* gene. Compared with the control cattle, the transgenic cattle exhibited enhanced resistance to virulent *M. bovis*^13^. Hence, understanding the molecular mechanism underlying Sp110 actions is expected to promote the breeding of TB-resistant transgenic animals and assist with the discovery of new therapies against TB. In this study, we determined in detail the transcriptomic changes in the host in relation to *Mtb* infection and modulation of such changes by Sp110 using transcriptome analysis in cultured murine macrophages. We found that Sp110 modulates the expression of cytokines, chemokines, and various genes involved in regulating mycobacterial growth, thereby enhancing the innate immune responses to *Mtb* infection. Sequencing of small RNA molecules revealed that Sp110 regulates expression of the miRNAs associated with immune responses. Furthermore, we investigated the role played by Sp110-induced BCL2 modifying factor (Bmf) in macrophage apoptosis. Our findings provide new insights into the mechanism of Sp110-mediated macrophage resistance to mycobacterium.

## Results

### *Mtb* infection activates macrophage immune responses

**Figure 1:**
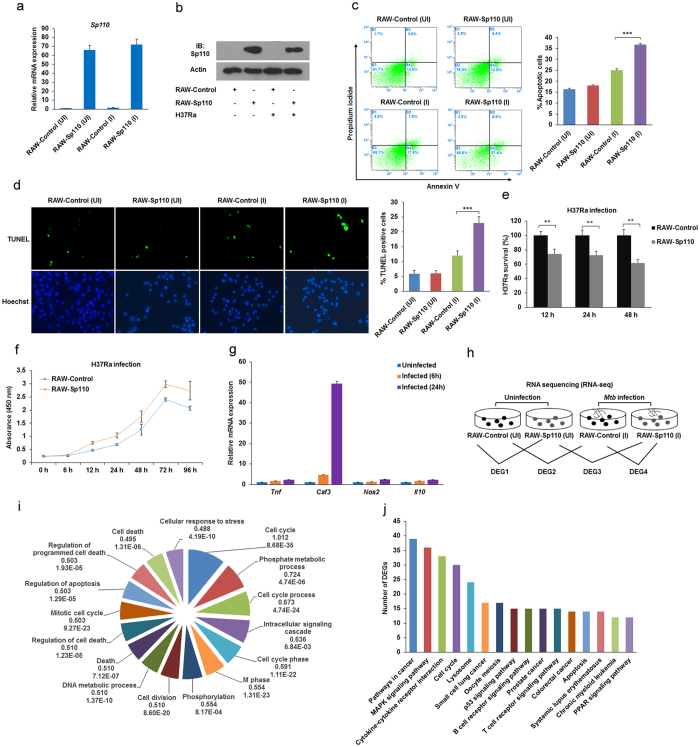
Transcriptional changes in mouse macrophages in response to *Mtb* infection. RAW-Control and RAW-Sp110 cells were infected with H37Ra at a multiplicity of infection (MOI) of 5:1 for 24 h, and Sp110 expression was determined by qPCR (**a**) or immunoblotting (**b**). RAW-Control and RAW-Sp110 cells were infected with H37Ra (MOI 5:1) for 24 h. Apoptotic cells were evaluated by Annexin-V staining followed by flow cytometric analysis (**c**) or a TUNEL assay (**d**). (**e**) RAW-Control and RAW-Sp110 cells were infected with H37Ra. Intracellular mycobacterial burden was determined at the time points indicated by a CFU assay. (**f**) The viabilities of the H37Ra-infected RAW-Control and RAW-Sp110 cells were determined by a cell proliferation assay. (**g**) RAW-Control and RAW-Sp110 cells were infected with H37Ra, and Tnf, Csf3, Nos2 and Il10 expression was determined by qPCR at the time points indicated. Data represent the mean ± SD of three independent experiments. **p < 0.01; ***p < 0.001. (**h**) Differentially expressed genes (DEGs) were screened as the groups indicated. UI, uninfected cells; I, H37Ra infected cells. (**i**) Gene Ontology (GO) analysis of H37Ra-regulated genes. Pie chart of the enriched biological processes generated from DEGs of DEG2. The top-ranked GO terms are presented according to the enriched gene counts, and the values below each term represent the ratio of DEGs versus the total gene set for each functional category and the P value. (**j**) Kyoto Encyclopedia of Genes and Genomes (KEGG) pathway analysis of H37Ra-regulated genes. The top-ranked pathways were plotted according to the DEG numbers. The complete GO and KEGG analysis data are shown in Table S2. The full-length blots are shown in Supplementary Figure 1.

To investigate the effect of Sp110 expression on the gene expression profiles of macrophages in response to *Mtb* infection, we transduced RAW264.7 cells with lentiviruses encoding Sp110 to establish a cell line stably overexpressing Sp110 (RAW-Sp110); RAW264.7 cells transduced with empty lentiviruses were used as a control (RAW-Control). RAW-Control and RAW-Sp110 cells were infected with the *Mtb* H37Ra strain and uninfected cells served as the controls. At 24 h post-infection, the cells were harvested and Sp110 expression was examined by quantitative (q) PCR and immunoblotting. The qPCR and immunoblot results confirmed that Sp110 was expressed stably in RAW-Sp110 cells (Fig. 1a,b). However, we noted that the endogenous Sp110 protein in the RAW-Control cells was not detectable by immunoblotting with an antibody against Sp110 (Fig. 1b). We speculated that lack of detection of Sp110 protein in RAW264.7 cells might because low sensitivity of the antibody used. Next, we analysed the apoptotic rates of the uninfected and infected RAW-Sp110 cells, and the Annexin-V staining and terminal deoxynucleotidyl transferase-dUTP nick end-labelling (TUNEL) assay results showed that overexpression of Sp110 solely did not affect apoptosis in the macrophages. In contrast, the H37Ra-infected RAW-Sp110 cells had a significantly higher apoptotic rate than that of the infected RAW-Control cells (Fig. 1c,d). These results indicate that mycobacterium stimulation is required for the Sp110-mediated apoptotic pathway.

To evaluate RAW-Sp110 resistance to *Mtb*, we analysed the intracellular bacterial burden and cell viability of the H37Ra-infected RAW-Control and RAW-Sp110 cells. As expected, the bacterial burden of the RAW-Sp110 cells decreased significantly in comparison with the RAW-Control cells (Fig. 1e). Furthermore, the H37Ra-infected RAW-Sp110 cells exhibited higher viability than the infected RAW-Control cells (Fig. 1f). After determining that Sp110 overexpression enhances resistance to H37Ra in RAW264.7 cells, total RNA from the H37Ra-infected RAW-Control and RAW-Sp110 cells was extracted. The qPCR results revealed that the well-known *Mtb*-activated genes *Tnf*, *Csf3*, *Nos2* and *Il10* were upregulated at 6 h and 24 h post-infection, and the fold changes at 24 h were higher than at 6 h (Fig. 1g). Hence, RNA samples from the uninfected and 24 h post-infected RAW-Control and RAW-Sp110 cells were used for RNA sequencing (RNA-seq). An overview of the primary sequencing data is depicted in Supplementary Table S1. Following data processing and analysis, the differentially expressed genes (DEGs) (p < 0.05, fold change >1.5) were identified by calculating the expression of each gene in the sample groups as indicated in Fig. 1h. Inspection of the DEGs in Supplementary Table S2 revealed that H37Ra induced expression of a large number of genes. Compared with the uninfected RAW-Control cells, 1451 genes showed transcriptional changes in the H37Ra-infected RAW-Control cells, of which 576 were upregulated and 875 were downregulated (Supplementary Table S2). Table 1 shows the top 10 upregulated and downregulated DEGs between the H37Ra-infected and uninfected RAW-Control samples. Notably, many of the top-ranked DEGs have immune-related functions and these include, for example, the colony stimulating factor 3 (granulocyte) (*Csf3*), the interleukin 1 receptor antagonist (*Il1rn*), the chemokine (C-X-C motif) ligand 2 (*Cxcl2*), the interleukin 7 receptor (*Il7r*), the chemokine (C-C motif) ligand 2 (*Ccl2*), the kin of IRRE-like 3 gene (*Kirrel3*), and the tumour necrosis factor (ligand) superfamily, member 13b gene (*Tnfsf13b*). Moreover, we found that infection with H37Ra inhibited the expression of genes associated with apoptosis regulation, such as the chemokine (C-X3-C motif) receptor 1 gene (*Cx3cr1*) and *Bmf* (Table 1).

Functional annotation clustering by Gene Ontology (GO) revealed that the H37Ra-regulated genes are highly enriched for terms associated with the cell cycle, intracellular signalling cascade, regulation of cell death, cellular response to stress, and immune responses (Fig. 1i, Supplementary Table S2). Kyoto Encyclopedia of Genes and Genomes (KEGG) pathway analysis showed that the DEGs from DEG2 are highly enriched for cell cycle pathways, the MAPK signalling pathway, cytokine-cytokine receptor interaction, apoptosis, the B cell receptor signalling pathway and the T cell receptor signalling pathway (Fig. 1j, Supplementary Table S2). These results suggest that infection with *Mtb* H37Ra effectively activated the immune response and intracellular signal transduction in mouse macrophages.

### Regulation of gene expression by Sp110 in uninfected and *Mtb*-infected macrophages

**Figure 2:**
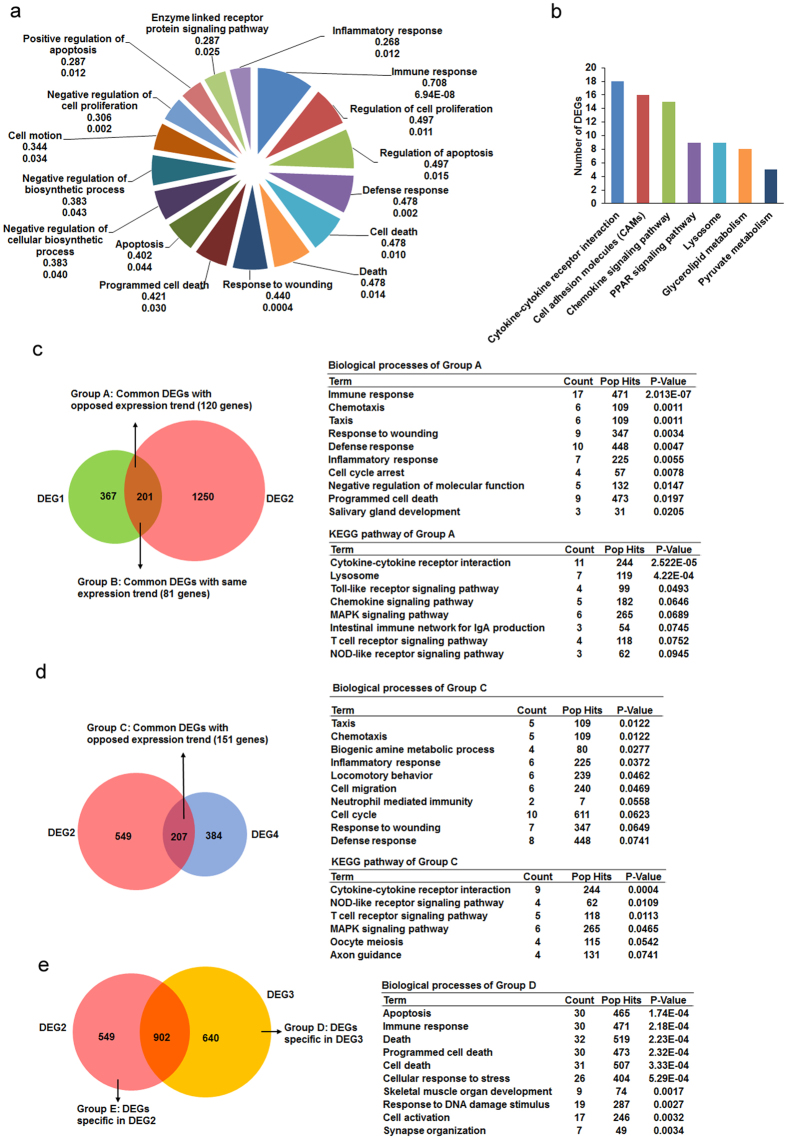
Functional annotation of Sp110-regulated genes. (**a**) GO analysis of Sp110-regulated genes. Pie chart of the enriched biological processes generated from the differentially expressed genes (DEGs) of DEG1. The top-ranked GO terms are presented according to the enriched gene counts, and the values below each term represent the ratio of DEGs versus the total gene set for each functional category and the P value. (**b**) KEGG pathway analysis of DEGs from DEG1. The top-ranked pathways were plotted according to the DEG numbers. (**c**) Comparison of DEGs from DEG1 and DEG2. The common DEG count between DEG1 and DEG2 with the same or opposite expression trend is shown in the left panel, and functional annotation of the group A DEGs is shown in the right panel. (**d**) Comparison of DEGs from DEG2 and DEG4. The common DEG count between DEG2 and DEG4 with an opposite expression trend is shown in the left panel, and functional annotation of these DEGs is shown in the right panel. (**e**) Comparison of DEGs from DEG2 and DEG3. The number of DEGs specific to DEG2 or DEG3 (left), and functional annotation of the DEGs specific to DEG3 (right), are provided. The complete GO and KEGG analysis data are shown in Tables S5–7.

The protein motifs in mouse Sp110 suggest its potential role in transcriptional regulation^8^. Hence, we investigated potential changes in the gene expression profiles of RAW-Sp110 and RAW-Control cells in the presence or absence of infection with H37Ra. In the uninfected cells, we found 568 genes with 1.5-fold or higher (p < 0.05) expression-level changes between RAW-Sp110 cells and RAW-Control cells, of which 204 were upregulated and 364 were downregulated by Sp110 (Supplementary Table S3). Of interest, *Mtb* infection resulted in more DEGs in the RAW-Sp110 cells than in the RAW-Control cells when compared with their uninfected counterparts. Transcriptional changes between the uninfected and H37Ra-infected RAW-Sp110 cells occurred for 1542 genes (p < 0.05) (Supplementary Table S4). Functional characterization of the DEGs from DEG1 according to GO terms revealed that the Sp110-regulated genes were highly enriched for terms associated with immune responses, regulation of cell proliferation, regulation of apoptosis, defence responses, cell death, responses to wounding and inflammatory responses, all of which are consistent with the physiological functions of Sp110 (Fig. 2a, Supplementary Table S3). The top-ranked pathways hit by Sp110-regulated genes were cytokine-cytokine receptor interactions, cell adhesion molecules, the chemokine signalling pathway, the PPAR signalling pathway, lysosome, glycerolipid metabolism, and pyruvate metabolism (Fig. 2b, Supplementary Table S3).

Comparison of DEG1 and DEG2 revealed that 120 genes (Group A) exhibited opposite expression patterns. These genes were enriched for GO terms relating to immune responses, chemotaxis, responses to wounding, defence responses, inflammatory responses, cell cycle arrest, and programmed cell death (Fig. 2c, Supplementary Table S5). Multiple immune response genes and apoptosis-related genes were upregulated by H37Ra but downregulated by Sp110, and vice versa; these genes include *Csf3*, *Ccl3*, *Tnf*, *Ccl2*, *Tlr13*, *Tnfrsf13b*, *Il1rn*, *Cxcl2*, *Ccl9*, *Myo1f*, *Acp5*, *Rsad2*, *Ccl4*, *Il10*, *Clec4e*, *Procr*, *Gp49a*, *Lilrb4*, *Relt*, *Sh3kbp1*, *Xaf1*, *Gadd45b*, *Niacr1*, *Bmf*, *Pdcd1* and *Phlda1* (Supplementary Table S5). Furthermore, comparison of DEG2 and DEG4 showed 151 genes with opposite expression patterns, and these genes were enriched for GO terms relating to chemotaxis, inflammatory responses, neutrophil-mediated immunity and responses to wounding (Fig. 2d, Supplementary Table S6). These data imply that Sp110 may reverse *Mtb-*induced gene expression, thereby reducing the adverse effects of infection.

Most importantly, we found that the *Mtb*-infected RAW-Sp110 cells (DEG3) harboured 640 DEGs not observable in the *Mtb*-infected RAW-Control cells (DEG2). The DEGs specific for DEG3 were enriched mainly in the biological processes of apoptosis, immune responses, cellular stress responses, responses to DNA damage stimulus and cell activation (Fig. 2e, Supplementary Table S7). In contrast, the top-ranked GO terms for DEGs specific for DEG2 were associated with the cell cycle, transcription, and cellular biosynthetic processes (Supplementary Table S7). Differences in the DEGs between DEG2 and DEG3 revealed Sp110-mediated signalling pathways following *Mtb* infection, and DEGs specific for DEG3 represent a possible transcriptional signature of Sp110-mediated macrophage resistance to *Mtb*. Taken together, the transcriptome analysis revealed that Sp110 regulates the transcription of genes involved in immune responses, defence responses, apoptosis and inflammatory responses, indicating that Sp110 activates the defence responses of macrophages to *Mtb* and mediates apoptosis via regulating protein-coding gene expression.

### Sp110 regulates the expression of cytokines and chemokines in mouse macrophages

**Figure 3:**
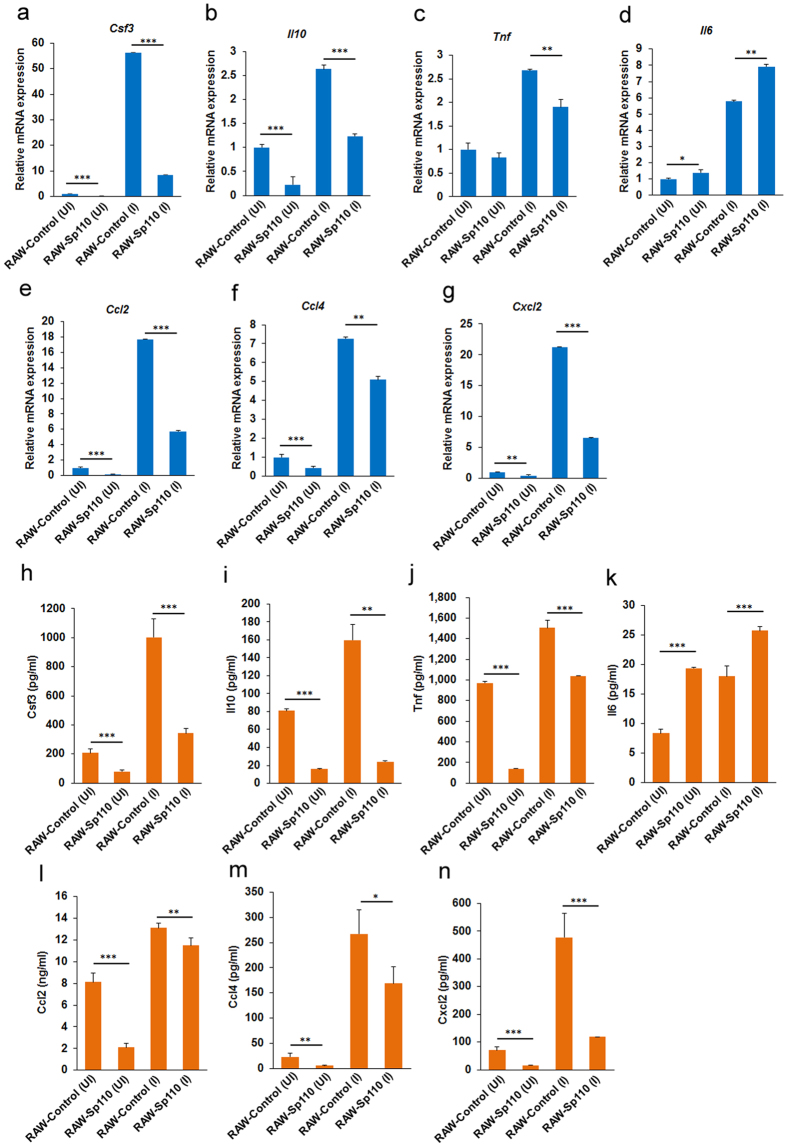
The regulatory effects of Sp110 and *Mtb* on cytokines and chemokines in mouse macrophages. (**a–g**) RAW-Control and RAW-Sp110 cells were infected with H37Ra at a multiplicity of infection (MOI) of 5:1 for 24 h. Expression of *Csf3*, *Il10*, *Tnf*, *Il6*, *Ccl2*, *Ccl4* and *Cxcl2* was determined by qPCR. Data are normalized to the uninfected RAW-control. (**h–n**) RAW-Control and RAW-Sp110 cells were infected with H37Ra (MOI 5:1) for 24 h. Secretion of Csf3, Il10, Tnf, Il6, Ccl2, Ccl4 and Cxcl2 was assessed by ELISA. UI, uninfected cells; I, infected cells. Data represent the mean ± SD of three independent experiments. *p < 0.05; **p < 0.01; ***p < 0.001, compared with controls.

Activation of innate immune mechanisms by the elevation of endogenous inflammatory cytokines and chemokines levels is crucial for clearance or containment of mycobacterial pathogens^14^. Indeed, *in vitro* infection of bovine alveolar macrophages with *Mtb* H37Rv and *M. bovis* significantly upregulated several innate immune genes (i.e., *TLR2*, *CCL4*, *IL1B*, *IL6* and *TNF*)^15^. Our sequencing data revealed that infection with H37Ra upregulated *Ccl4* and *Tnf*. Additionally, *Csf3* and *Il10* cytokine genes and *Ccl2* and *Cxcl2* chemokine genes were upregulated by H37Ra (Table 2). Surprisingly, we found that Sp110 upregulated *Il6* and downregulated *Csf3*, *Il10*, *Tnf*, *Ccl2*, *Ccl4* and *Cxcl2* in mouse macrophages (Table 2). Furthermore, the qPCR results confirmed that Sp110 overexpression inhibits *Csf3*, *Il10*, *Tnf*, *Ccl2*, *Ccl4* and *Cxcl2*, but enhances Il6 expression under both uninfected and H37Ra-infected conditions (Fig. 3a–g).

Next, we examined the cytokines and chemokines in the supernatants of uninfected and *Mtb*-infected RAW-Control and RAW-Sp110 cells. ELISA was performed to assess the concentrations of Csf3, Il6, Il10, Tnf, Ccl2, Ccl4 and Cxcl2. The secretion of Csf3, Il10 and Tnf cytokines decreased in the RAW-Sp110 cells when compared with the RAW-Control cells, whereas Il6 secretion increased (Fig. 3h–k). Moreover, the results showed that the secreted chemokines Ccl2, Ccl4 and Cxcl2 in RAW-Sp110 cells were lower than that of the RAW-Control cells (Fig. 3l–n). Therefore, we speculated that Sp110 may control mycobacterial burden via regulating *Mtb*-activated cytokines and chemokines, because these proinflammatory and anti-inflammatory genes are involved in innate immunity to *Mtb*^16^,^17^.

### Sp110 regulates the expression of genes involved in cell death and intracellular survival of *Mtb*

**Figure 4:**
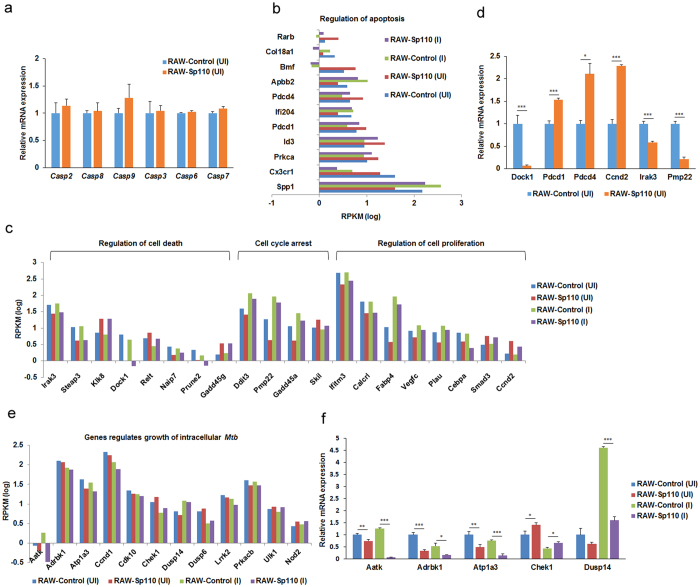
Sp110 regulates genes involved in regulating apoptosis and intracellular survival of *Mtb.* (**a**) RAW-Control and RAW-Sp110 cells were infected with H37Ra at a multiplicity of infection of 5:1 for 24 h and then expression levels of the genes indicated were determined by qPCR. (**b**) The RNA-seq levels of a selection of apoptosis regulators in uninfected or H37Ra-infected RAW-control and RAW-Sp110 cells. (**c**) The RNA-seq levels of selected genes associated with cell death regulation, cell cycle arrest, and cell proliferation regulation in uninfected or H37Ra-infected RAW-control and RAW-Sp110 cells. (**d**) Validation of DEGs by qPCR. (**e**) RNA-seq levels of the genes involved in regulating the intracellular growth of *Mtb* in uninfected or H37Ra-infected RAW-control and RAW-Sp110 cells. (**f**) Validation of the DEGs associated with intracellular growth of *Mtb* by qPCR. qPCR data are normalized to the uninfected RAW-Control and represent the mean ± SD of three independent experiments. *p < 0.05; **p < 0.01; ***p < 0.001, compared with controls.

Sp110 is known to inhibit the multiplication of intracellular pathogens and it switches a cell death pathway in the infected macrophages from necrosis to apoptosis^8^. Unexpectedly, the RNA-seq and qPCR data showed that the apoptotic initiator caspases (Caspase-2, -8, -9) and apoptotic effector caspases (Caspase-3, -6, -7)^18^ underwent no significant transcriptional changes in the Sp110 overexpressed cells (Fig. 4a). However, we found that Sp110 regulated the transcription of apoptosis-related genes; for example, the proapoptotic genes *Pdcd1*, *Pdcd4* and *Bmf* were upregulated by Sp110 (Fig. 4b). Furthermore, transcriptional changes were also observed for genes associated with cell death regulation (*Irak3*, *Steap3*, *Klk8*, *Dock1*, *Relt*, *Naip7*, *Prune2*, *Gadd45g*), cell cycle arrest (*Ddit3*, *Pmp22*, *Gadd45a*, *Skil*) and cell proliferation regulation (*Ifitm3*, *Calcrl*, *Fabp4*, *Vegfc*, *Plau*, *Cebpa*, *Smad3*, *Ccnd2*) between the RAW-Control and RAW-Sp110 cells (Fig. 4c). The qPCR results support Sp110 upregulation of *Pdcd1*, *Pdcd4* and *Ccnd2*, and downregulation of *Dock1*, *Irak3* and *Pmp22* (Fig. 4d).

Some host genes play pivotal roles in combating infection and the intracellular survival of *Mtb*. Knockdown of Aatk, Adrbk1, Atp1a3 and Dusp14 in mouse macrophages resulted in a significant decrease in the intracellular mycobacterial load, while knockdown of Chek1 and Wee1 led to a marked increase in the mycobacterial level^19^. In addition, the nucleotide-binding oligomerization domain protein, NOD2, is important for regulating the host response to *Mtb* and bacillus Calmette–Guérin infection in human macrophages^20^. Interestingly, our RNA-seq results revealed that Sp110 downregulates *Aatk*, *Adrbk1*, *Atp1a3* and *Dusp14* and upregulates *Chek1* and *Nod2* under both uninfected and infected conditions (Fig. 4e), and the qPCR results confirmed that Sp110 regulates these host-dependent survival factors for intracellular *Mtb* (Fig. 4f), indicating that Sp110 controls *Mtb* survival and growth by regulating the expression of genes involved in bactericidal responses.

### Sp110 regulates miRNA expression in macrophages

**Figure 5:**
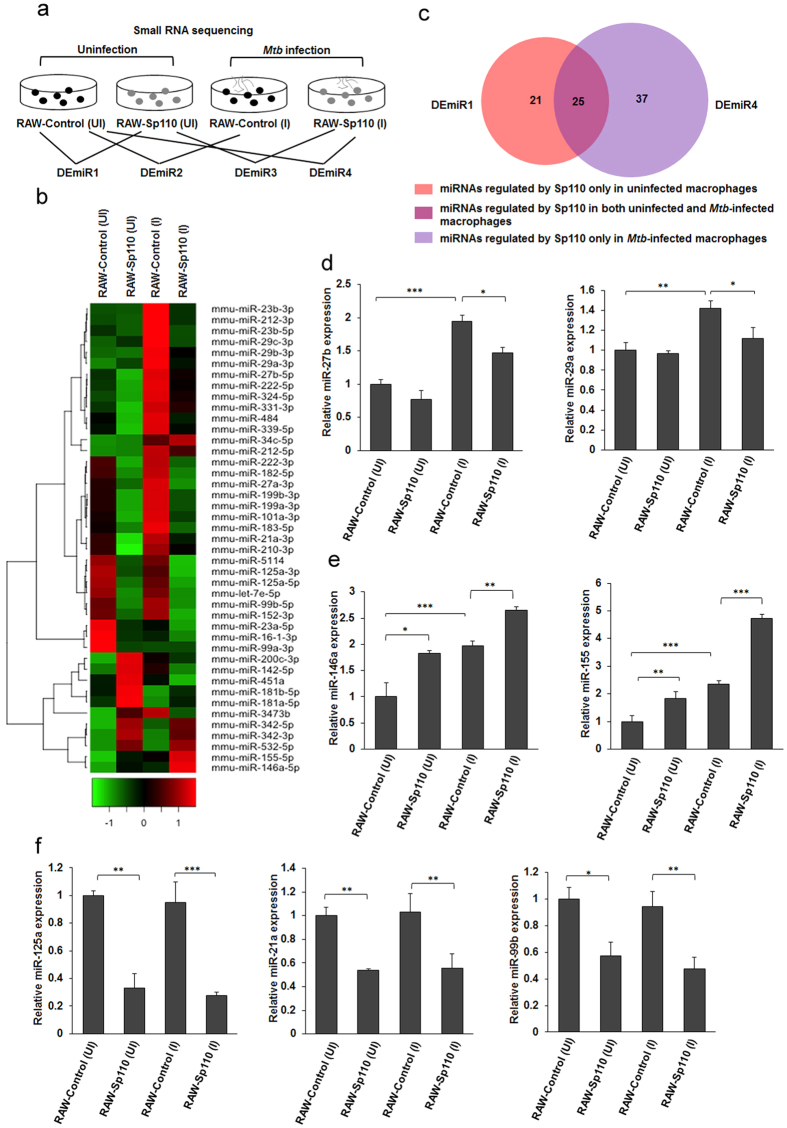
Sp110 regulates miRNA expression in uninfected and *Mtb*-infected macrophages. (**a**) The experimental scheme used for studying the miRNA expression profiles of RAW-Control and RAW-Sp110 cells in response to *Mtb* infection. Small RNA sequencing was performed on the uninfected and the H37Ra-infected RAW-Control and RAW-Sp110 cells at a multiplicity of infection of 5:1 for 24 h. Differentially expressed miRNAs were screened according to the groups indicated. DEmiR, differentially expressed miRNA. (**b**) Differences in the miRNA profiles of the H37Ra-infected RAW-Control/RAW-Sp110 cells and their uninfected counterparts were determined by small RNA sequencing. Selected DEmiRs are presented in a heat map. Green and red indicate low and high expression, respectively. (**c**) Venn diagrams showing the numbers of DEmiRs in uninfected RAW-Sp110 cells and in *Mtb*-infected RAW-Sp110 cells, with reference to basal expression levels in the uninfected RAW-Control cells. (**d**) Sp110 inhibits *Mtb*-induced miR-27b and miR-29a expression. (**e**) Sp110 enhances miR-146a and miR-155 expression. (**f**) Sp110 inhibits miR-125a, miR-21a and miR-99b expression. Expression of each miRNA in the uninfected and the H37Ra-infected RAW-Control and RAW-Sp110 cells was determined by qPCR. UI, uninfected cells; I, H37Ra infected cells. qPCR data represent three independent experiments. Error bars indicate mean ± SD (n = 3), *p < 0.05; **p < 0.01; ***p < 0.001, compared with controls.

Recent studies have reported that miRNA regulates innate and adaptive immune responses to intracellular pathogens^4^,^21^, and is involved in the macrophage apoptosis response to *Mtb*^22^. To investigate whether miRNAs are involved in Sp110-mediated macrophage resistance to *Mtb*, small RNA sequencing was performed to identify Sp110-regulated miRNAs in the presence or absence of H37Ra infection, after which the differential miRNA expression was analysed (Fig. 5a). We found that Sp110 affected the expression of 46 miRNAs in the uninfected macrophages, of which 15 miRNAs were significantly upregulated and 31 miRNAs were significantly downregulated (p < 0.05) (Fig. 5b, Supplementary Table S8). Notably, miR-155 and miR-200c, which have been reported to be capable of inducing apoptosis^22^,^23^, were upregulated by Sp110. Moreover, Sp110 significantly inhibited the miR-99b-let-7e-125a cluster, miR-152, miR-21a, miR-23a and miR-27b (Fig. 5b, Supplementary Table S8). H37Ra infection led to robust changes in miRNA expression, upregulating 41 miRNAs and downregulating 11 miRNAs. Consistent with previous studies, *Mtb* induced miR-155, miR-29a and miR-29c expression, and these miRNAs inhibited immune-related gene responses to *Mtb* infection (Fig. 5b, Supplementary Table S8). Additionally, miR-16-1, miR-23a, miR-25 and miR-99a levels decreased in macrophages in response to H37Ra infection. These results confirm previous findings that intracellular pathogens can alter miRNA expression to modulate host immune responses^4^,^24^.

Comparison of differentially expressed miRNA (DEmiR) DEmiR1 and DEmiR4 revealed that Sp110 regulates 21 miRNAs in uninfected macrophages only. In fact, miR-101, miR-210, miR-21a, miR-27b, miR-331, miR-339, miR-484 and miR-8106 were downregulated by Sp110, while miR-142, miR-181a, miR-200c, miR-3473b and miR-451a were upregulated by Sp110 (Fig. 5c, Supplementary Table S9). Moreover, 37 miRNAs were regulated by Sp110 in H37Ra-infected macrophages only (i.e., miR-106b, miR-1198, miR-139, miR-151, miR-25, miR-146a and miR-34c; Fig. 5c, Supplementary Table S9). Of note, in both uninfected and *Mtb*-infected macrophages, Sp110 induced miR-155, miR-342, miR-3470a and miR-532, but inhibited let-7e, miR-1249, miR-125a, miR-132, miR-152, miR-16-1, miR-182, miR-183, miR-23a, miR-28a, miR-5114, miR-99a and miR-99b. These results suggest that Sp110 is able to regulate miRNA expression in macrophages; furthermore, *Mtb* infection also plays critical roles in modulating Sp110-regulated miRNAs.

qPCR analysis was performed to verify the small RNA sequencing data. The results confirmed that expression of miR-27b and miR-29a in the RAW-Control induced by H37Ra was downregulated significantly in the H37Ra-infected RAW-Sp110 cells (Fig. 5d). It has been shown that upregulation of miR-146a and miR-155 enhances the clearance of intracellular mycobacteria in macrophages^21^,^25^. Our data showed that miR-146a and miR-155 were upregulated by Sp110 under uninfected and *Mtb*-infected conditions (Fig. 5e). We also noted that expression of miR-99b, miR-125a and miR-21a was inhibited by Sp110 (Fig. 5f). Considering the significance of these miRNAs in regulating immune responses to mycobacteria, the results suggest that Sp110 protects host cells from *Mtb* infection by regulating host miRNA expression, particularly *Mtb*-regulated miRNA expression.

### Sp110-upregulated Bmf induces macrophage apoptosis

**Figure 6:**
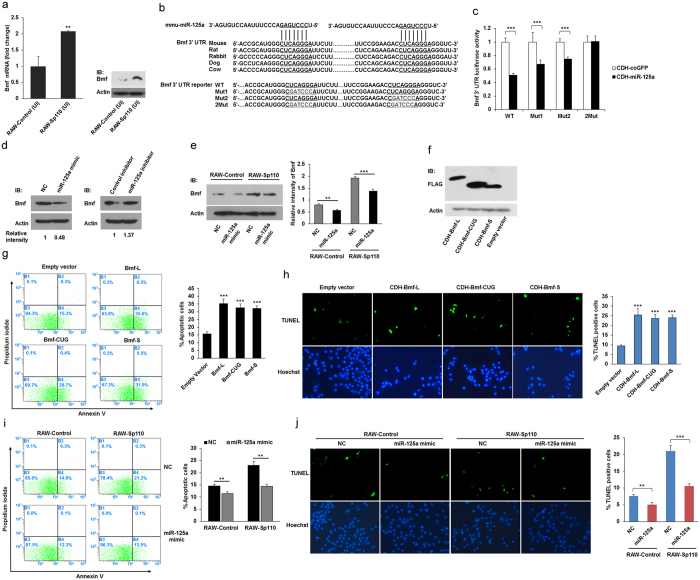
The effect of Sp110-upregulated Bmf on macrophage apoptosis. (**a**) The mRNA and protein levels of the *Bmf* gene in uninfected RAW-Control and RAW-Sp110 cells were determined by qPCR and immunoblots, respectively. (**b**) Conserved miR-125a binding sites (underlined) in the 3′ UTR of the *Bmf* mRNA (top), and mutations introduced into the reporter constructs (bottom). (**c**) Relative luciferase activity in the lysates of HEK293T cells cotransfected with a reporter construct encoding the wild-type (WT) or mutated (Mut) *Bmf* 3′ UTR and the miRNA expression construct pCDH-miR-125a for 48 h. (**d**) Endogenous Bmf protein expression in RAW264.7 cells transfected with a mimic or inhibitor of miR-125a-5p for 24 h was determined by immunoblotting. (**e**) RAW-Control and RAW-Sp110 cells were transfected with the miR-125a mimic for 24 h, after which Bmf expression was examined by immunoblotting. (**f**) RAW264.7 cells were transduced with lentiviruses encoding different isoforms of Bmf for 30 h, after which Bmf expression was detected by immunoblotting. Apoptosis of RAW264.7 cells transduced with lentiviruses encoding different isoforms of Bmf for 30 h was determined by Annexin-V staining followed by flow cytometric analysis (**g**) or a TUNEL assay (**h**). RAW-Control and RAW-Sp110 cells were transfected with the miR-125a mimic, and 6 h after transfection, cells were infected with H37Ra at a multiplicity of infection of 5:1 for 24 h. Cell apoptosis was determined by Annexin-V staining followed by flow cytometric analysis (**i**) or a TUNEL assay (**j**). Data represent three independent experiments. *p < 0.05; **p < 0.01; ***p < 0.001. The full-length blots are shown in Supplementary Figure 2.

Our integrative analysis of Sp110-regulated mRNA and miRNA data revealed that the apoptosis-inducing gene, *Bmf*^26^, is a potential target of miR-125a. Consistently, we found that overexpression of Sp110 significantly downregulated miR-125a in RAW264.7 cells (Fig. 5f), but upregulated *Bmf* (Fig. 6a). Targetscan database prediction showed that the 3′ UTR of *Bmf* mRNA consists of two conserved miR-125a-5p binding sites (Fig. 6b). To confirm that miR-125a-5p suppresses Bmf expression through interaction with the *Bmf* 3′ UTR, we constructed a miRNA expression vector (pCDH-miR-125a) and *Bmf* 3′ UTR reporter vectors. HEK293T cells were cotransfected with pCDH-miR-125a and a wild-type or mutated *Bmf* 3′ UTR reporter vector. The luciferase assay results showed that overexpression of miR-125a significantly decreased the luciferase activity of the wild-type *Bmf* 3′ UTR reporter; however, the two binding sites in the mutated reporter vector did not respond to the increased level of miR-125a (Fig. 6c). Moreover, we found that transfection of RAW264.7 cells with the miR-125a mimic inhibited endogenous expression of Bmf. In contrast, transfection of the miR-125a inhibitor enhanced Bmf expression in the RAW264.7 cells (Fig. 6d). Furthermore, the RAW-Control and RAW-Sp110 cells were transfected with the miR-125a mimic, and the immunoblot result showed that the miR-125a mimic decreased the Bmf protein level in both RAW-Control and RAW-Sp110 cells (Fig. 6e). These results suggest that miR-125a interacts with the *Bmf* 3′ UTR to inhibit Bmf expression, and that Sp110 upregulates the expression of Bmf by downregulating miR-125a.

The mouse *Bmf* mRNA sequence encodes three Bmf isoforms of different lengths using alternative translation start sites (named Bmf-L, Bmf-CUG and Bmf-S), and all three isoforms can induce apoptosis of U2OS cells^26^. To determine whether upregulation of Bmf alone is sufficient to induce apoptosis of mouse macrophages, we constructed lentiviral expression plasmids for mouse Bmf-L, Bmf-CUG and Bmf-S. RAW264.7 cells were transduced with lentiviruses encoding Bmf-L, Bmf-CUG or Bmf-S, while cells transduced with empty lentiviruses were used as a control. The results showed that the three Bmf isoforms were expressed correctly in RAW264.7 cells (Fig. 6f). Furthermore, overexpression of Bmf-L, Bmf-CUG or Bmf-S increased the apoptotic rates of the macrophages compared with that of the control (Fig. 6g,h). Moreover, we found that Sp110-mediated apoptosis decreased in *Mtb*-infected macrophages transfected with the miR-125a mimic (Fig. 6i,j). Collectively, these results confirm that overexpression of Bmf is able to induce apoptosis of mouse macrophages, and suggest that Sp110 downregulates miRNA expression to upregulate the protein level of apoptosis-related genes as an alternative mechanism of Sp110-mediated macrophage apoptosis.

## Discussion

The mouse *Sp110* gene plays critical roles in regulating innate immunity to intracellular pathogens and mutations in its human homolog are associated with immunodeficiency diseases^8^,^27^. We have shown previously that overexpression of mouse Sp110 enhances host cell resistance to virulent strains of *M. bovis* both *in vitro* and *in vivo*^9^,^13^. To date, however, the molecular mechanism of Sp110-mediated macrophage resistance to *Mtb* remains unknown, and the downstream genes involved in Sp110-mediated pathways are unexplored. Most recently, high-throughput sequencing and microarray technologies have been introduced to analyse the mammalian host response to mycobacterial pathogens, the results of which have greatly enhanced scientific understanding of the interactions occurring between host species and pathogens^2^,^28^,^29^,^30^,^31^,^32^. Because macrophages are the main host cells for *Mtb*, efficient genetic manipulation of them is required to investigate innate immunity to this pathogen, but achieving this is especially difficult in primary macrophages. The mouse RAW264.7 cell line is used widely in *Mtb* studies because of its macrophage-like characteristics; hence, we used RAW264.7 cells as a model to identify Sp110 regulated genes in response to avirulent *Mtb* H37Ra. Our RNA-seq results confirmed that avirulent H37Ra activated the immune response in RAW264.7 cells. We noted that the top-ranking DEGs from the H37Ra-infected RAW264.7 cells were enriched for biological processes and pathways that differ from other *Mtb* strains infected cells^2^,^28^,^29^, this might be caused by differential virulence in the *Mtb* strains and differences in host cell features in these studies.

The effect of Sp110 on transcription in the uninfected and *Mtb*-infected macrophages was analysed and the Sp110-regulated genes were found to be associated predominantly with immune responses, regulation of apoptosis and defence responses. Moreover, we found that the Sp110-regulated genes were enriched in biological processes related to the wounding response and the inflammatory response. KEGG analysis revealed that Sp110 plays critical roles in cytokine-mediated and chemokine-mediated signalling. Differential expression of such genes not only confirmed the role of Sp110 in apoptotic pathway switching, but also revealed some new and potential functions for Sp110, such as lesion healing and inflammation control. Comparison of Sp110-regulated genes and H37Ra-regulated genes found that 120 genes had opposing directions of expression associated with immune responses, responses to wounding, defence responses, inflammatory responses, and programmed cell death, raising the possibility that Sp110 mediates macrophage resistance to *Mtb* by antagonizing *Mtb*-induced gene expression. Furthermore, the DEGs from DEG2 and DEG3 exhibited significant differences from one another. The DEGs exclusive to DEG3 were highly enriched in GO terms for apoptosis, immune responses, cellular responses to stress and cell activation, whereas those specific to DEG2 were classified mainly as other biological processes. In addition, we noted that the DEGs in uninfected RAW-Sp110 cells (DEG1) were not enriched for the term “cellular response to stress”, whereas the DEGs in DEG2 and DEG3 matched this term, and more DEGs in DEG3 matched this term than those in DEG2. Previous studies have shown that the endoplasmic reticulum stress response is involved in *Mtb*-induced apoptosis^33^,^34^,^35^. Therefore, our data indicate that Sp110 induces apoptosis following *Mtb* infection, and that the *Mtb*-induced cellular stress response is essential for the Sp110-mediated apoptotic pathway.

Following infection with *Mtb*, alveolar macrophages and dendritic cells recruit monocytes, NK cells, T cells and neutrophils to form a granuloma that partitions off the infected cells, thereby controlling *Mtb* replication and preventing *Mtb* from escaping into the surrounding tissues^36^. The balance of inflammatory response of cells within the granuloma is pivotal to limiting *Mtb* growth and controlling excessive inflammation, both of which are responsible for the outcome of *Mtb* infection^37^. Our results show that *Mtb* infection largely increased the transcription and protein levels of *Csf3*, *Il6*, *Il10*, *Tnf*, *Ccl2*, *Ccl4* and *Cxcl2*. However, Sp110 markedly suppressed expression of these inflammatory factors, except for Il6. It has been reported that Csf3 is involved in maintaining neutrophil populations^38^. At the granuloma, Il10 is a critical regulator of lesion outcome, and decreased Il10 levels increase the number of sterile lesions, while increasing Il10 levels promote bacterial persistence by limiting the early antimicrobial response and preventing lesion sterilization^39^. Hence, we speculate that reduced expression of Il10 in RAW-Sp110 cells contributed to mycobacteria clearance. Tnf is a critical host defence molecule against TB, and moderate Tnf levels are beneficial for controlling mycobacterial growth, however, low and high Tnf levels lead to necrosis and *Mtb* release^40^. The Ccl2 chemokine has been shown to participate in granuloma formation and to protect against *Mtb*, while Ccl4 has been described as an inhibitor of bacilli growth^41^,^42^. Nevertheless, an increased Ccl2 level was shown to be associated with TB severity^16^. Collectively, our findings suggest that Sp110 maintains *Mtb*-regulated cytokines and chemokines at optimal levels to preserve the balance of mycobacterial burden and tissue lesions, thereby enhancing host resistance to *Mtb*.

Sp110 plays crucial roles in regulating apoptosis in *Mtb*-infected macrophages. Unexpectedly, the apoptotic initiator and effector caspases produced no significant transcriptional changes in cells overexpressing Sp110. However, Sp110 enhanced the transcription of the proapoptotic gene *Bmf*^26^, and altered several other apoptotic regulators of expression. Moreover, antiapoptotic *Bcl2* decreased in RAW-Sp110 cells following *Mtb* infection. Hence, we speculated that Sp110 turns on the apoptotic pathway by affecting the cellular apoptotic regulators rather than the apoptotic initiators or effectors of expression. Successful infection of macrophages by *Mtb* depends on effective weakening of the diverse microbicidal responses mounted by the host cell, which is achieved by targeted perturbations of the host cellular signalling machinery. Based on this principle, Jayaswal and colleagues identified host genes that regulate intracellular survival of *Mtb* through siRNA screening. They found that silencing of Aatk, Adrbk1, Atp1a3 and Dusp14 reduced *Mtb* survival to various extents, while silencing Chek1 resulted in an increase in the mycobacterial burden^19^. The present study showed that Sp110 inhibits Aatk, Adrbk1, Atp1a3 and Dusp14, and promotes Chek1 expression in mouse macrophages, thus providing the molecular foundations for the growth inhibition of intracellular mycobacteria by Sp110.

miRNAs, a class of non-coding RNAs, play important regulatory roles across a wide variety of biological processes^24^. Recent studies have explored the role of miRNAs in regulating host immunity to specific *Mtb*^5^,^21^,^22^,^43^,^44^,^45^,^46^. Our data revealed that Sp110 affects miRNA expression in both uninfected and *Mtb*-infected cells. The miR-99b-let-7e-125a cluster and miR-132, both of which modulate the innate host defence response to *Mtb*^5^,^46^,^47^,^48^, were inhibited significantly by Sp110. However, Sp110 upregulated miR-155, miR-342, miR-3470a, miR-532 and miR-690. The significance of miR-155 in the interaction between the host and *Mtb* has been well documented^21^,^22^, but further studies are needed to elucidate the functions of the other Sp110-regulated miRNAs. Interestingly, we found that Sp110 regulates *Mtb*-induced miRNA expression. The presence of Sp110 inhibited the induction effects of H37Ra on miR-27b and miR-29a, while enhanced H37Ra induced miR-146a and miR-155 expression. Upon infection with *Mtb*, miR-29 levels increased and suppressed the immune responses to intracellular pathogens via targeting the cytokine, IFN-γ^4^, whereas overexpression of miR-146a and miR-155 enhanced the mycobacteria killing ability of the host cell through different mechanisms^21^,^25^. Thus, these findings suggest that Sp110 modulates miRNA expression in macrophages, thereby subsequently regulating host immunity to infection with *Mtb*.

Bmf, a Bcl2 family member and proapoptotic protein, triggers apoptosis by binding to antiapoptotic Bcl-2 proteins^49^. Our data showed that Sp110 upregulated Bmf by inhibiting miR-125a and forced expression of Bmf induces macrophage apoptosis. In contrast, H37Ra significantly suppressed Bmf expression, suggesting that Bmf may be a key regulator of Sp110-mediated host immunity against *Mtb*. Overall, using an avirulent strain of *Mtb* and the RAW264.7 murine macrophage cell line, our data revealed the transcriptional basis of Sp110-mediated macrophage resistance to TB. As expected, Sp110 was found to regulate the expression of genes involved in apoptosis-related biological processes. Moreover, our results indicate that Sp110 has some unexplored functions relating to the regulation of innate immunity to *Mtb*, because Sp110 altered the expression of cytokines, chemokines and other genes involved in the intracellular survival of *Mtb*, thereby influencing inflammation and *Mtb* growth. This study also identified Sp110-regulated miRNAs from uninfected and infected macrophages. Collectively, the present study indicates that Sp110 protects the host against *Mtb* by modulating a complex network of mRNAs and miRNAs.

## Methods

### Cell and mycobacterial culture, and infection

HEK293T cells were cultured in DMEM medium supplemented with 10% FBS. RAW264.7 cells purchased from the American Type Culture Collection (ATCC) were maintained in RPMI1640 medium supplemented with 10% FBS. All cells were maintained at 37 °C and 5% CO2 in a humidified incubator. *M. tuberculosis* strain H37Ra (ATCC 25177) was grown in Middlebrook 7H9 broth medium supplemented with 10% OADC (Becton, Dickinson and Company, Franklin Lakes, NJ). Cells were infected at a multiplicity of infection of 5 bacteria per cell (MOI 5:1). After 6 h of incubation, the infected cells were washed 6 times with RPMI1640 to remove any extracellular bacteria, and then incubated in fresh medium for a further 18 hours.

### CFU assay

To assay intracellular bacterial burden of macrophages, cells were infected with H37Ra for 6 h and then washed with RPMI1640 to remove extracellular bacteria. The infected cells were incubated for the indicated time and then lysed in PBS with 0.05% SDS, and intracellular bacteria were plated on Middlebrook 7H10 agar plates supplemented with OADC and incubated for 3 weeks at 37 °C to determine the colony number.

### Generation of constructs and stably transfected cells

The *Sp110* ORF sequence was amplified by PCR using cDNA from C57BL/6 mouse lung. The lentiviral expression vector pCDH-*Sp110* was generated by inserting *Sp110* ORF sequence into the pCDH-MCS-T2A-Puro-MSCV vector (System Biosciences, Mountain View, CA). To generate lentiviral vector expressing Bmf, the ORF sequences encode isoforms of Bmf were amplified by PCR using different upstream primers and a common downstream primer, and a FLAG tag was fused at the N-terminus, the resulting fragments were cloned into pCDH-MCS-T2A-Puro-MSCV, respectively. To construct the pCDH-miR-125a vector, DNA sequence of the primary miR-125a was amplified and inserted into the pCDH-CMV-MCS-EF1-copGFP vector (System Biosciences). To construct the luciferase reporter plasmids, the 3′ UTR sequence of mouse Bmf mRNA was amplified by RT-PCR and cloned into psiCHECK-2 (Promega, Madison, WI). The mutations in the 3′ UTR of mouse Bmf mRNA was introduced by overlap extension PCR. To generate stably expressed cells, RAW264.7 cells were transduced with viral supernatants collected from the HEK293T cells transfected with lentiviral constructs. After 48 h transduction, puromycin (5 μg/ml) was added to the dishes for additional 5 d to screen stably transfected cells, and expression of target genes was verified by immunoblotting.

### Luciferase reporter assays

Luciferase assays were performed using a Dual-Luciferase Reporter Assay System (Promega, Madison, WI) according to the manufacturer’s instructions. Briefly, HEK293T cells were cotransfected with luciferase reporter and pCDH-miR-125a using Lipofectamine 2000 Reagent (Invitrogen, Carlsbad, CA). After 48 h, transfected cells cells were lysed in 1× passive lysis buffer for 15 min with shaking. Firefly luciferase activity in cell lysates was measured on a VICTOR X5 Multilabel Plate Reader (PerkinElmer, Cetus, Norwalk) and normalized to *Renilla* luciferase.

### Enzyme-Linked Immunosorbent Assay (ELISA)

At 24 h after infection, supernatants from uninfected and *Mtb*-infected macrophages were collected by centrifugation. Levels of Il6, Il10, Tnf (R&D Systems Inc., Minneapolis, MN), Csf3, Ccl2, Ccl3, and Cxcl2 (Cloud-Clone Corp., Houston, TX) were determined in triplicate by ELISA following manufacturer’s instructions.

### RNA isolation, RNA-seq and small RNA sequencing

RAW-Control and RAW-Sp110 cells were seeded on 6-well plate, and then infected with H37Ra (MOI 5:1) for 24 h, uninfected RAW-Control and RAW-Sp110 cells were used as controls. For each treatment, three independent experiments were conducted to prepare the samples. Total RNA was extracted using Trizol reagent (Life technologies, Carlsbad, CA) following the manufacturer’s instructions. RNA integrity was checked by an Agilent Bioanalyzer 2100 system (Agilent technologies, Santa Clara, CA). Qualified total RNA of each sample was divided into two copies, one for RNA-seq and the other one for Small RNA sequencing experiment. For RNA-seq and Small RNA sequencing, the total RNA from three independent experiments of each treatment was pooled respectively. RNA-seq and Small RNA sequencing was carried out by Beijing Genomics Institute (BGI, Shenzhen, China) on an Illumina HiSeq 2000 sequencing system (Illumina, San Diego, CA). Library preparation, sequencing, and quality and quantity control of raw sequence were conducted as described^50^,^51^. Sequencing data are submitted to NCBI SRA (PRJNA279114, PRJNA279232).

### Bioinformatics analysis of sequencing data

Read counts were normalized by the Reads per Kilobase of feature per Million Mapped Reads (RPKM) method^52^. Following normalization, the fold-change between treatment and control sample was calculated as: fold-change = log2 (treatment/control), the p-value was calculated using the formula described previously^53^. In addition, differentially expressed genes were filtered on the criteria that each gene in each sample had an RPKM greater than zero which excluded genes expressed at low levels in all samples (read count <10). To better understand potential functions of the differentially expressed genes, GO and KEGG pathway analysis were performed using the online tool of the Database for Annotation, Visualization, and Integrated Discovery (DAVID).

### Validation of differentially expressed mRNAs and miRNAs by quantitative PCR (qPCR)

The miRNA expression was validated by poly (A)-tailed qPCR. Total RNA was extracted from macrophages using Trizol reagent, and then 2 μg of RNA was reverse-transcribed to cDNA using miScript II RT Kit (Qiagen GmbH, Hilden, Germany) according to the manufacturer’s instructions. qPCR was performed using SYBR Premix Ex Taq II (Tli RNaseH Plus) (Takara, Dalian, China) on an StepOne Plus PCR system (Applied Biosystems, Foster City, CA). For each reaction, 1 μL of diluted cDNA (equivalent to 10 ng of total RNA) was mixed with 10 μL of 2 × SYBR Premix. The final volume of 20 μL was achieved by the addition of 5 pmol of the forward primers and the universal reverse primers. For qPCR experiments of mRNA, reverse-transcription was performed using a SYBR PrimeScript RT reagent Kit (Takara, Dalian, China). All the primers used are provided in Supplementary Table S10. The specificity of the primer amplicons was examined by the analysis of a melting curve. The comparative Ct method was employed for quantification of target miRNA and mRNA expression. The relative expression of miRNA was normalized to small nuclear RNA (Rnu6) expression and the relative expression of mRNA was normalized to *GAPDH* expression.

### Immunoblotting (IB)

Cells were lysed with RIPA buffer (Pierce, Rockford, IL) according to the manufacturer’s instructions. The protein concentration of lysates was quantified using BCA Protein Assay Reagent (Pierce). The protein samples were separated by SDS-PAGE followed by immunoblotting as described^50^. Primary antibodies used were Bmf (Cell Signaling Technology, Danvers, MA), Sp110 (Santa Cruz Biotechnology, Inc., Dallas, TX), FLAG (Sigma, Saint Louis, MO), and Actin (TransGen Biotech Co., Ltd., Beijing, China). Secondary antibodies were purchased from Beyotime (Jiangsu, China).

### Transient transfection of miRNA mimic and inhibitor

RAW264.7 cells were transfected with 50 nM control mimic, miR-125a mimic, control inhibitor or miR-125a inhibitor (Shanghai GenePharma Co., Ltd., Shanghai, China) using Lipofectamine 2000 reagent (Invitrogen). Total protein was extracted 48 h after transfection followed by immunoblotting.

### Apoptosis assays

Cell apoptosis was assessed by binding of Annexin V-Alexa Fluor 488 (Molecular Probes, Eugene, OR). Cells were incubated in Annexin binding buffer (10 mM HEPES, 140 mM NaCl and 2.5 mM CaCl2) and stained with 10 μl Annexin V-Alexa Fluor 488 for 20 min, followed by counterstaining with 1 μg/ml propidium iodide for 15 min. Subsequently, cells were washed twice with cold PBS and fixed with 1% paraformaldehyde for 30 min, and then washed once with cold PBS. Binding of Annexin V-Alexa Fluor 488 and PI was detected by BD FACS Calibur flow cytometer (BD Biosciences, San Jose, CA). Apoptosis was also examined by the terminal deoxynucleotidyl transferase dUTP nick-end labeling (TUNEL) assay using the Click-iT Plus TUNEL Assay Kit (Molecular Probes). Labeled cells were analyzed by fluorescence microscope and counted.

### Cell viability assay

Cell viability was evaluated with a TransDetect® Cell Counting Kit (TransGen Biotech Co., Ltd.) according to the manufacturer’s instructions. Briefly, RAW-Control and RAW-Sp110 cells were seeded on 96-well plates and infected with H37Ra (MOI 5:1). The absorance of CCK8 solution incubated cell supernants was measured at 450 nm using an Epoch microplate spectrophotometer (BioTek Instruments, Winooski, USA). Growth curves were constructed using the mean values of six replicates.

### Statistical analysis

The data were represented as the mean ± SD and were analyzed using the Student’s t-test. A value of p < 0.05 was considered significant.

## Additional Information

**How to cite this article**: Wu, Y. *et al.* The Transcriptional Foundations of Sp110-mediated Macrophage (RAW264.7) Resistance to *Mycobacterium tuberculosis* H37Ra. *Sci. Rep.* **6**, 22041; doi: 10.1038/srep22041 (2016).

## Supplementary Material

Supplementary Information

Supplementary Table S1

Supplementary Table S2

Supplementary Table S3

Supplementary Table S4

Supplementary Table S5

Supplementary Table S6

Supplementary Table S7

Supplementary Table S8

Supplementary Table S9

## Figures and Tables

**Table 1:** The top10 upregulated and downregulated genes (*p* < 0.05) for H37Ra-infected versus control RAW264.7 cells at 24 h post infection as ranked by fold-change.

gene Symbol	gene name	log2 fold change	P-value
Csf3	colony stimulating factor 3 (granulocyte)	5.91	0
Gpnmb	glycoprotein (transmembrane) nmb	4.90	1.35E-167
Il1rn	interleukin 1 receptor antagonist	4.89	0
Atp6v0d2	ATPase, H+ transporting, lysosomal V0 subunit D2	4.54	2.78E-141
Cxcl2	chemokine (C-X-C motif) ligand 2	4.36	0
Il7r	interleukin 7 receptor	4.26	7.85E-68
Anpep	alanyl (membrane) aminopeptidase	4.09	7.14E-155
Ccl2	chemokine (C-C motif) ligand 2	3.90	0
Gdf15	growth differentiation factor 15	3.69	7.93E-50
Tgm2	transglutaminase 2, C polypeptide	3.66	1.66E-35
Cx3cr1	chemokine (C-X3-C motif) receptor 1	−3.00	2.17E-185
Fgd2	FYVE, RhoGEF and PH domain containing 2	−2.67	4.36E-33
Myh7b	myosin, heavy chain 7B, cardiac muscle, beta	−2.33	6.83E-11
Bmf	BCL2 modifying factor	−2.29	8.82E-16
Kirrel3	kin of IRRE like 3	−2.08	8.38E-14
Trem3	triggering receptor expressed on myeloid cells 3	−2.02	2.47E-10
Pi16	peptidase inhibitor 16	−2.02	8.16E-19
Gpr155	G protein-coupled receptor 155	−1.90	4.03E-07
Tnfsf13b	tumor necrosis factor (ligand) superfamily, member 13b	−1.85	1.06E-05
Sla	src-like adaptor	−1.85	7.93E-13

**Table 2:** Expression changes of cytokines and chemokines in uninfected and H37Ra-infected RAW-Control and RAW-Sp110 cells.

Gene Symbol	RAW-Control(UI)	RAW-Control (I)	RAW-Sp110 (UI)	RAW-Sp110 (I)
Ccl2	29.153	435.668	2.420	151.043
Ccl3	1348.953	9382.225	566.321	9243.169
Ccl4	239.837	1249.591	86.141	1233.978
Ccl9	119.386	217.558	68.528	273.859
Fgr	12.580	11.940	5.817	5.162
Cxcl2	90.446	1862.397	40.173	1185.504
Cxcl14	1.455	1.224	3.816	2.732
Tiam2	1.981	1.710	1.221	0.728
Tiam1	3.117	2.656	1.518	0.927
Cx3cr1	39.565	4.962	19.188	2.383
Grk5	2.536	2.633	1.556	1.541
Gng7	2.025	1.709	1.183	1.095
Cxcl10	7.078	8.669	3.617	11.977
Csf3	6.335	379.808	1.321	231.021
Flt1	29.910	22.514	5.399	4.869
Tnf	353.745	813.921	210.534	615.101
Tnfrsf13b	4.718	1.951	9.201	7.693
Il10	5.202	17.008	2.642	22.531
Il6	0.781	4.283	1.104	5.573
Il1rn	17.580	521.424	6.533	339.028
Vegfc	8.278	12.304	5.184	8.742
Relt	4.824	2.833	7.349	4.711
Il10ra	13.819	8.530	31.525	15.196
Il2rg	27.924	33.665	42.811	42.324

The normalized expression value of each gene (RPKM) for each sample was shown.
