# Supplementary material for: The Transcriptional Foundations of Sp110-mediated Macrophage (RAW264.7) Resistance to Mycobacterium tuberculosis H37Ra
Source: Sci Rep. 2016 Feb 25;6:22041. doi: 10.1038/srep22041 (PMC4766572; doi:10.1038/srep22041)
Supplement: Supplementary Information [file srep22041-s1.pdf]

The Transcriptional Foundations of Sp110-mediated Macrophage (RAW264.7) Resistance to *Mycobacterium tuberculosis* H37Ra

Yongyan Wu[1,2], Zekun Guo[1,2], Kezhen Yao[1,2], Yue Miao[1,3], Shuxin Liang[1,2], Fayang Liu[1,2], Yongsheng Wang[1,2], and Yong Zhang[1,2,*]

1 College of Veterinary Medicine, Northwest A&F University, Yangling 712100, Shaanxi, China

2 Key Laboratory of Animal Biotechnology, Ministry of Agriculture, Northwest A&F University, Yangling 712100, Shaanxi, China

3 Innovative Experimental College, Northwest A&F University, Yangling 712100, Shaanxi, China

* Corresponding author: Yong Zhang (zhylab@hotmail.com), Tel: 86-2987080085

Supplementary Figure 1. **Full-length blots of Figure 1 (b) in the main text.**

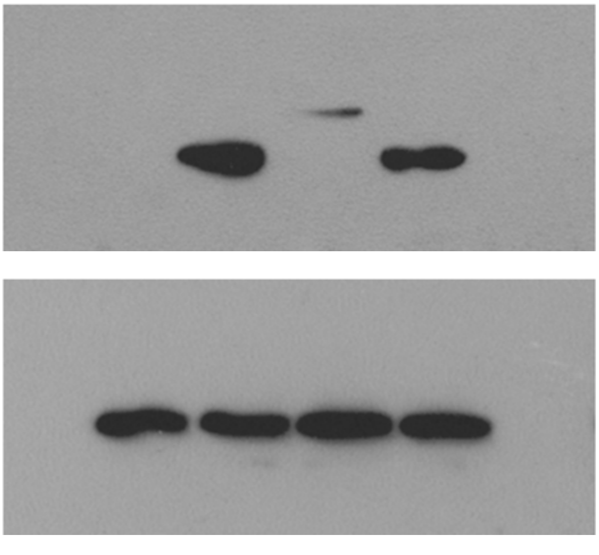

Supplementary Figure 2. **Full-length blots of Figure 6 (a), (d), (e) and (f) in the main text.**

Figure 6 (a)

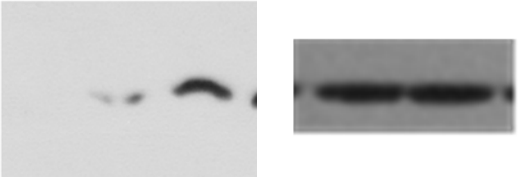

Figure 6 (d)

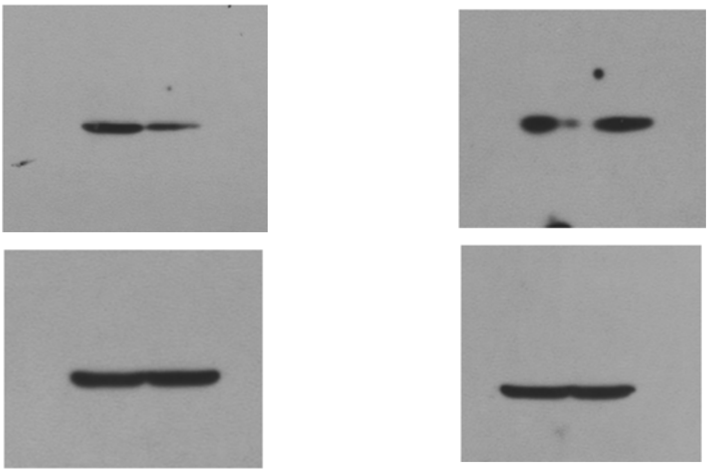

Figure 6 (e)

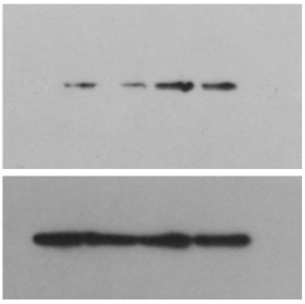

Figure 6 (f)

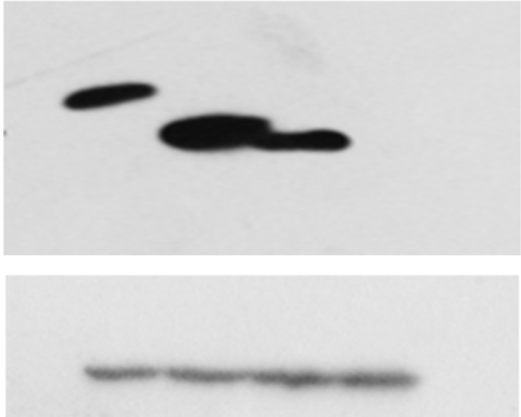

Supplementary Table 10. **Primer sequences for qPCR analysis.**

Gene name	Forward primer (5'- 3')	Reverse primer (5'-3')
Bmf	GGAGCGGGCGTATTTTGGAA	ACACTCGATTGGGAAGAAGGG
Dock1	AGTACGGCGTGGCCTTTTAC	GACGGTTTCATGTTGCCCTTT
Il10	GCTCTTACTGACTGGCATGAG	CGCAGCTCTAGGAGCATGTG
Ccl2	TTAAAAACCTGGATCGGAACCAA	GCATTAGCTTCAGATTTACGGGT
Aatk	GGGAGGTATGAGTGTGGTGG	TAGATCCCCATTCGTGGTGTC
Adrbk1	GCGCCAGCAAGAAGATCCT	GCAGAAGTCCCGGAAAAGCA
Atp1a3	TCTCAGATGTGTCCGTTCTTCT	TGGAAAGAGAGTGAAAGGCAAG
Dusp14	TTGCTCAGATCACCTCCTCTC	AGTACAGTCTAATGGGGGCAT
Chek1	GTTAAGCCACGAGAATGTAGTGA	GATACTGGATATGGCCTTCCCT
Csf3	ATGGCTCAACTTTCTGCCCAG	CTGACAGTGACCAGGGGAAC
Cxcl2	CCAACCACCAGGCTACAGG	GCGTCACACTCAAGCTCTG
Irak3	AGGCCAAGTTAAGACCCACG	ACCTCAGACTGGCTGCATTC
Pdcd4	CGAGGGGATCCTCAAGGAAGA	TTTCCGCAGTCGTCTTTTGG
Pdcd1	TCATGAGTGCCCTAGTGGGT	AGGGAGCTCTGGTGTCTTCT
Ccnd2	AGTCCCGACTCCTAAGACCC	GAAGTCGTGAGGGGTGACTG
Pmp22	CATCGCGGTGCTAGTGTTG	AAGGCGGATGTGGTACAGTTC
Ccl4	TTCCTGCTGTTTCTCTTACACCT	CTGTCTGCCTCTTTTGGTCAG
Il6	GCCTTCTTGGGACTGATGCT	TGCCATTGCACAACTCTTTTCT
Tnf	CCCTCACACTCACAAACCAC	ATAGCAAATCGGCTGACGGT
Nos2	GGTGAAGGGACTGAGCTGTT	ACGTTCTCCGTTCTCTTGCAG
Casp2	CACAGACTCCTGCGTAGTGG	TGTCTCATCTCCACGACATGC
Casp3	GAGCTTGGAACGGTACGCTA	GCGAGATGACATTCCAGTGC
Casp6	GAGGTGTCGACTTCAAGCCA	CCGACAGGCCTGGATGATAA
Casp7	CCTCTGGGACTTTTGCTTTCA	CTGATCATCGGTCATCGTTCC
Casp8	CGGAGGCATTTCTGTCCCCTA	AGAGCTGTAACCTGTGGCCG
Casp9	ACCTTCCCAGGTTGCCAATG	AGCCTGCCCCAGTTCAAAAT
Gapdh	GTGTTCCTACCCCCAATGTGT	ATTGTCATACCAGGAAATGAGCTT
miR-125a	TCCCTGAGACCCTTTAACC	The reverse primer for qPCR experiments of miRNAs is a universal primer provided by the miScript II RT Kit (QIAGEN).
miR-21a	GGGTAGCTTATCAGACTGATGTTGA	
miR-27b	AGAGCTTAGCTGATTGGTGAAC	
miR-29a	ACTGATTTCTTTTGGTGTTCAG	

miR-146a	GGTGAGAACTGAATTCCATG	
miR-155	TTAATGCTAATTGTGATAGGGGT	
miR-99b	CACCCGTAGAACCGACCTTGC	
Rnu6	TGCGCAAGGATGACACGC	
